# Domesticated Animal Biobanking: Land of Opportunity

**DOI:** 10.1371/journal.pbio.1002523

**Published:** 2016-07-28

**Authors:** Linn F. Groeneveld, Sigbjørn Gregusson, Bernt Guldbrandtsen, Sipke J. Hiemstra, Kristian Hveem, Juha Kantanen, Hannes Lohi, Lina Stroemstedt, Peer Berg

**Affiliations:** 1 NordGen—the Nordic Genetic Resource Center, Ås, Norway; 2 BioBank AS, Hamar, Norway; 3 Aarhus University, Aarhus, Denmark; 4 Centre for Genetic Resources, the Netherlands (CGN), Wageningen University and Research Centre, Wageningen, the Netherlands; 5 Department of Public Health, Norwegian University of Science and Technology (NTNU), Trondheim, Norway; 6 Natural Resources Institute Finland (Luke), Helsinki, Finland; 7 Department of Environmental and Biological Sciences, University of Eastern Finland, Kuopio, Finland; 8 Research Programs Unit, Molecular Neurology, and Department of Veterinary Biosciences, University of Helsinki, Helsinki, Finland; 9 SLU Biobank, Department of Animal Breeding and Genetics, Swedish University of Agricultural Sciences (SLU), Uppsala, Sweden

## Abstract

In the past decade, biobanking has fuelled great scientific advances in the human medical sector. Well-established domesticated animal biobanks and integrated networks likewise harbour immense potential for great scientific advances with broad societal impacts, which are currently not being fully realised. Political and scientific leaders as well as journals and ethics committees should help to ensure that we are well equipped to meet future demands in livestock production, animal models, and veterinary care of companion animals.

In the last decade, human biobanking has emerged as an important driver of scientific activities, and biobanks are indisputably an invaluable resource for all types of research aimed at improving public health. The combination of accessible and well-characterized biological samples of various types linked with a multitude of associated data is driving scientific discoveries at unprecedented speed and making previously unthinkable lines of research a reality [1,2].

Unfortunately, biobanking of animal samples is by far less well-established. In March 2015, *Nature* published an article, titled “Inside the first pig biobank,” describing a newly established biobank of porcine samples to be used in studying human diabetes and hailing it as a pioneering effort in animal biobanking [3]. A PubMed search confirmed that in comparison to human biobanking there appears to be negligible activity in the animal biobanking sector. Searching titles, abstracts, and keywords with the search keys “biobank,” “biobanking,” “genebank,” and “gene bank” and limiting the results to publication dates in 2015, only 9 of 498 search results referred to animal biobanks (see S1 Data). This apparent lack of activity in the animal biobanking sector is also reflected in a 2015 editorial of *Biopreservation and Biobanking*, the official journal of the International Society for Biological and Environmental Repositories (ISBER), which caters to biobanks of any species. The authors conclude that even though there has been increasing participation from the non-human biobanking sector in the annual ISBER meetings, there is still a pronounced lack of submissions to the journal pertaining to non-human biobanking, and human biobanking issues continue to dominate ISBER activities [4]. The roadmap of the European Council’s European Strategy Forum on Research Infrastructures (ESFRI) reveals that there are projects under way involving human (Biobanking and BioMolecular Resources Research Infrastructure [BBMRI]), marine (European Marine Biological Resource Centre [EMBRC]), microbial (Microbial Resource Research Infrastructure [MIRRI]), and mouse model (Infrafrontier) biobanks, with general animal biobanks starkly missing on that list [5].

Naturally, some non-human biobanks storing animal samples, amongst others, do exist. The most active are likely the natural history collections, because they have the intrinsic task to collect, catalogue, and store specimens. The Global Genome Biodiversity Network (GGBN), established in 2011, acts as an umbrella organisation for biodiversity repositories and aims to establish standards and best practices as well as increase sample accessibility through its data portal [6]. A search of the most common domesticated animal species (cattle, sheep, goat, pig, horse, chicken, and dog) yielded only 13 records in the GGBN member repositories.

However, some domesticated animal biobanks and less formalized sample collections can be found. Their hosting institutions range from veterinary hospitals, zoos, breeding and diagnostics companies, national farm animal genetic resource gene banks, to research institutes and universities. Depending on their purpose, the stored types of samples vary greatly and range from healthy tissue samples, diseased pathogenic tissue samples, DNA, and RNA to reproductive materials. An example of a well-established physical non-human biobanking infrastructure serving a university is the Swedish University of Agricultural Sciences’ (SLU) Biobank (http://www.slu.se/slubiobank). This biobank also offers a data portal for increasing the visibility and accessibility of non-human sample collections no matter where they are stored. This data portal would be redundant if all samples, together with their associated data, were stored in established biobanks that ensured the visibility of their samples through a network such as GGBN. In contrast, the European Genebank Network for Animal Genetic Resources (EUGENA), coordinated by the European Regional Focal Point on Animal Genetic Resources (http://www.rfp-europe.org), is an emerging networking activity specifically targeting only national farm animal genetic resource collections [7]. These disparate examples demonstrate that there is a lack of a unified and generalized approach to sample collections in the domesticated animal sector.

Nonetheless, there are numerous examples of how different disciplines and stakeholders, and ultimately the general public, have already benefitted from the availability of biobanked domesticated animal samples.

Even though the pig biobank was commended as a pioneering effort [3], there are in fact a number of biobanks that accommodate animal models for the study of human disease. The domestic dog, for example, with its unique population history, breed structure, and hundreds of spontaneous genetic conditions has proven to be an excellent model for gene mapping in simple and complex disorders [8]. Targeted and effective breeding programs over the past 150 years have created hundreds of distinct breeds that form genetic isolates with reduced genetic heterogeneity. This simplifies genetic studies because fewer susceptibility loci with higher impact contribute to complex disease and allow genetic breakthroughs with smaller study cohorts as compared to the corresponding human conditions [9].

The annotation of the canine genome facilitated a rapid evolution of genomic tools and development of several canine biobanks across the continents [10]. Collectively, these biobanks house hundreds of thousands of DNA samples and tissue specimens for hundreds of conditions with medical relevance to humans. Importantly, many canine biobanks maintain active collaborative networks with the breeder community and dog fanciers as well as veterinary clinics and hospitals for patient recruitment and health updates.

Besides playing an instrumental role for human health, biobanked animal samples heavily impact developments in food production and the sustainable management of the world’s finite resources. Biobanks in animal breeding, often referred to as gene banks, were initially established with the advent of new reproductive techniques, such as artificial insemination, and typically stored semen and embryos. These biobanks recently played a critical role in the swift implementation of genomic selection, which uses genome-wide SNP markers to predict the genetic merit of breeding individuals [11,12]. The efficient use of genomic selection requires large reference panels of individuals whose genetic values are known with high confidence. In cattle breeding, these are bulls with large numbers of offspring with recorded performance data, such as milk yield. Genomic selection could only be implemented so swiftly and successfully because DNA or semen samples from a large number of bulls were available from cattle breeding company biobanks, and these samples could be linked to performance records of the respective bulls’ offspring. This technology was first adopted by the dairy industry and can potentially result in a 60%–120% increase in the rate of genetic gain. Together with advanced genotyping and reproductive technologies, genomic selection has the potential to increase genetic improvement both in often neglected traits, such as feed efficiency and fertility, and in traits that only recently have become of interest, such as methane output in ruminants or adaptation to climate change [12]. Improvements in these traits are of great interest for ensuring global food security and sustainable management of our limited resources. Without the availability of the gene bank samples, as well as associated performance data records, this transformation would have taken decades, if it had happened at all.

Biobanks also play an integral part in worldwide conservation efforts to counteract the well-documented loss of genetic diversity in production animals [13,14]. Slowly, the general perception that these repositories are only to be used in emergencies and as a last resort is changing. In 2012, the USDA National Animal Germplasm Program, for example, harboured more than 700,000 gamete and tissue samples from over 18,000 animals representing more than 130 breeds. From this repository, samples from more than 3,300 animals had been requested and distributed for use in research and industry by 2012. The applications included quantitative trait locus (QTL) studies, assessment of genetic distances, cryobiology research, generation of an experimental research line, reduction of inbreeding, and re-introduction of genotypic combinations lost in current production populations [14]. Samples from rare and endangered breeds are also finding use in research and development of the leading breeding companies. For example, in the Netherlands, a consortium of university and dairy industry partners genotyped samples from rare local cattle breeds to gain insight into the genetic background of milk fatty acid composition. Genomic-assisted introgression could ultimately be used to introduce favourable alleles found in the rare breeds into more widely used breeds.

Biobanked samples also played an important role in fighting a viral infection, infectious pancreatic necrosis (IPN), which is common in farmed fish. This virus can lead to rates of >90% mortality in farmed Atlantic salmon, which, therefore, poses a threat to animal welfare and aquaculture industries. In 2008, a major QTL for IPN-resistance was detected in Atlantic salmon. Already, a year later, AquaGen, which supplies about 55% of Atlantic salmon eggs used commercially in Norway, was employing marker-assisted selection to produce IPN-resistant fish. This swift implementation of the QTL in marker-assisted selection was only possible due to the availability of biobanked samples collected in a challenge test in 2005 [15].

In addition to combatting disease in animals, biobanked domestic animal samples also play a crucial role in fighting emerging infectious diseases that are often zoonotic, meaning that they can be transmitted between vertebrate animals and humans. Having access to samples of species that act as reservoirs of a disease greatly facilitates the work of public health responders during infectious disease outbreaks [16]. In this context, the collection and traceable link of associated samples, such as parasites, pathogens, and other microbiota, to their parent sample becomes especially important.

We are convinced that these examples leave no doubt that biobanked animal samples hold great potential both for advancing human and animal health and welfare as well as securing future food production. Furthermore, the recent advent of cost-efficient gene modification technologies [17] envisages many production, performance, and health applications in livestock and companion animals and further adds interest in animal biobanks.

When examining the causes for the low levels of activity in large-scale domesticated animal biobanking, both in regard to the establishment or use of existing physical biobanking infrastructures as well as overarching data portals, a number of hypotheses come to mind. The industries connected to domesticated animal biobanking, such as livestock and companion animal production and veterinary care, are dwarfed by the healthcare industry, so monetary incentives would presumably play a much smaller role. Legislation may have acted as a driver in the formalization and shaping of biobanks and differential legislation regarding the handling, storage, and sharing of human versus animal biosamples, and associated data may thus have led to disparate developments. It is moreover conceivable that the community around domesticated animal biobanking is more fragmented and consists of more diverse stakeholders (academic, non-profit, industrial) than the human biobanking community, which could explain the absence of large-scale cooperative umbrella projects. Moreover, there may be greater difficulties in drafting material transfer agreements for reproductive materials than for other types of samples.

We will only be able to exploit the full potential if we, in parallel with human and biodiversity biobanking, tackle the challenges of standardized sampling, processing, and storage, sample visibility and accessibility, standardized codes for diagnoses, collection and storage of associated data with the possibility for updates, as well as ethical and regulatory issues. Here, it is advisable that the domesticated animal sector ensures full compatibility with and relies on existing initiatives wherever feasible. Especially important in this context is to ensure a link between samples and associated phenomic and genomic data, such as derived sequence data. To achieve agreement on standards, both in terms of sample processing and storage and sample visibility and accessibility, actors from veterinary hospitals, zoos, breeding and diagnostics companies, national farm animal genetic resource gene banks, research institutes, universities, and policymakers need to join forces. This is where we momentarily see a lack of coordinated efforts.

To respond to these challenges and to ensure that we are well equipped to meet future demands in livestock production, animal models, and veterinary care of companion animals, we propose that scientific and political leaders need to (i) acknowledge the inadequacy of the current situation, (ii) create opportunity and support for the establishment of an international research infrastructure for animal biobanking, and (iii) motivate academic and industrial stakeholders to develop and coordinate biobanks based on lessons learned from human and biodiversity biobanking.

In Europe, the European Council’s ESFRI could play a leading role in the establishment of a domesticated animal biobanking network, including best practices, direly needed standards, and a common ontology. In a landscape analysis of European research infrastructures, the 2016 ESFRI roadmap acknowledges a gap in the agricultural and bio-economy sector and explicitly lists livestock facilities including gene banks [5]. While an increase in activities regarding biobanking of farm animal genetic resources is certainly relevant, we consider this not to be far-reaching enough. A step in the right direction would be to begin with compiling information on all existing animal biobanks, analogous to BBMRI’s catalogue for European human biobanks [18], which currently contains information on 340 biobanks (http://www.bbmriportal.eu/).

Moreover, ethics committees should require the storage of samples and associated data in formalized biobanks for the approval of scientific experiments. Similarly, journals should apply the same standard to samples and associated data, as they currently apply to molecular data, in terms of storage in formalized repositories prior to publication.

## Supporting Information

S1 Data PubMed search. A PubMed search was carried out on January 14, 2016. Titles, abstracts, and keywords were searched with the search keys "biobank," "biobanking," "genebank," and "gene bank," and results were limited to publication dates in 2015.
(XLSX)

## Abbreviations

BBMRI: Biobanking and BioMolecular Resources Research Infrastructure
EMBRC: European Marine Biological Resource Centre
ESFRI: European Council’s European Strategy Forum on Research Infrastructures
EUGENA: European Genebank Network for Animal Genetic Resources
GGBN: Global Genome Biodiversity Network
IPN: infectious pancreatic necrosis
ISBER: International Society for Biological and Environmental Repositories
MIRRI: Microbial Resource Research Infrastructure
QTL: quantitative trait locus
SLU: Swedish University of Agricultural Sciences
